# Riding the Highs and Lows of the Conduction System Pacing Wave—Our Experience

**DOI:** 10.3390/jcdd12050164

**Published:** 2025-04-22

**Authors:** Hooi Khee Teo, Yi Yi Chua, Julian Cheong Kiat Tay, Xuanming Pung, Jonathan Wei Sheng Ong, Germaine Jie Min Loo, Eric Tien Siang Lim, Kah Leng Ho, Daniel Thuan Tee Chong, Chi Keong Ching

**Affiliations:** Department of Cardiology, National Heart Centre Singapore, Singapore 169609, Singaporeeric.lim.t.s@singhealth.com.sg (E.T.S.L.); ching.chi.keong@singhealth.com.sg (C.K.C.)

**Keywords:** conduction system pacing, stylet-driven leads, lumenless leads

## Abstract

Conduction system pacing started with His bundle pacing (HBP) and then rapidly switched gears into left bundle branch pacing (LBBP). We describe our center’s experience with LBBP using either lumenless leads (LLLs) or stylet-driven leads (SDLs). Patients who were admitted to two tertiary centers between 1 April 2021 and 30 June 2024 and met the guidelines for pacing were recruited and prospectively followed up. A total of 124 patients underwent permanent pacemaker (PPM) implantation using the LBBP technique with a mean follow-up of 19.7 ± 13.3 months. In total, 90 patients were implanted with LLLs and 34 with SDLs. There was no significant difference in the procedural time and final paced QRS duration, but fluoroscopy time was significantly longer in the SDLs (26.2 ± 17.7 min vs. 17.5 ± 13.0 min, respectively, *p* = 0.026). The on-table impedance values were also significantly higher in the LLLs, and this persisted throughout the follow-up. There were no differences in the rates of complications. The success of conduction system pacing implantation with SDLs and LLLs is comparable with reasonable safety and reliable outcomes. Good pre-implant patient selection will contribute to improved outcomes.

## 1. Introduction

The seed of conduction system pacing (CSP) was first introduced in 1970 [1], but it was not until the year 2000 that Deshmukh et al. [2] described the technique of His bundle pacing (HBP). With the idea of providing physiological pacing, a series of patients with chronic atrial fibrillation and dilated cardiomyopathy underwent HBP and showed some echographic improvements in cardiac function. Less than 20 years later, Huang et al. described the first case of successful left bundle branch pacing (LBBP) for a patient with heart failure and a left bundle branch block (LBBB) [3]. Since then, a wave of CSP implants has rapidly swept across continents with the incorporation of the technique becoming mainstream practice. Guidelines have scrambled to stay relevant, and as such, the EHRA clinical consensus statement on CSP implantation was published in April 2023 [4]. HBP is the most physiological form of pacing; however, it has faced issues including unpredictable long-term lead performance, low sensing values, and an inability to significantly correct bundle branch blocks [5]. On the other hand, LBBP implants show excellent sensing comparable to that of conventional right ventricular pacing. They are often able to fully correct underlying bundle branch blocks, have excellent thresholds, and lead to even greater improvements in ejection fraction (EF) as compared to conventional cardiac resynchronization (CRT) implants [5].

CSP was first performed using the lumenless Medtronic SelectSecure 3830 lead (LLL) that has a 1.8 mm fixed active helix. In recent years, existing conventional stylet-driven leads (SDLs) like the Biotronik Solia S 60 and the Tendril STS have been successfully used for LBBP as well. However, SDLs and LLLs do have significant differences in their design and structure [6], and it is important that these are considered when selecting patients for the most suitable type of lead.

We aim to describe our center’s experience of performing LBBP using the various sheaths and both LLLs and SDLs, including differences in technicality, complications, and the stability of lead parameters at follow-up.

## 2. Methods

Patients who were admitted to National Heart Centre Singapore (NHCS) and Sengkang General Hospital (SKH) between April 2022 and June 2024 and met the indications for pacing were considered for LBBP and prospectively followed up. LBBP was also considered for patients in whom attempts for a coronary sinus lead during cardiac resynchronization therapy (CRT) implantation were unsuccessful. Patients < 18 years old were excluded, and all patients provided informed consent as per institutional review board (IRB) requirements. This study was designed as a prospective, observational study to review our center’s experience with conduction system pacing.

The implantation procedure was performed by 6 operators at either of the 2 sites, and LBBP capture was confirmed as per the EHRA 2023 consensus statement [4]. The physician decided on the types of leads to be implanted—SDLs vs. LLLs. Leads and sheaths from three different companies were used—the Medtronic SelectSecure 3830 LLL delivered through the C315His sheath, the Biotronik Solia S60 SDL delivered through the Selectra 3D sheath, and the Abbott Tendril STS SDL delivered either through the Agilis HisPro steerable catheter or the Locator at the later stage. Patients were also differentiated to have true LBBP versus left ventricular septal pacing (LVSP) and deep septal pacing (DSP) based on the criteria described in the EHRA 2023 consensus statement [4]. As per the literature, the term left bundle area pacing (LBBAP) includes both LBBP and LVSP.

### Data Collection and Analysis

Data for patient demographics, comorbidities, indications for pacing, left ventricular ejection fraction (LVEF), and ECG characteristics were collected. Procedure-related information including the type of lead, lead parameters, number of attempts, immediate complications, and duration of fluoroscopy and procedure time was documented. Pacing parameters on post-procedure day 1, 3 months, and the latest available date were recorded.

In patients with a high-grade atrioventricular block (AVB) or bundle branch block (BBB) pattern, AV delays were programmed and optimized to achieve the narrowest paced QRS. In patients in whom the LBBP was implanted for CRT purposes, the output of the RV defibrillation lead was programmed to subthreshold. In patients who had sinus node dysfunction and no indication for CRT to reduce heart failure admissions, minimization of ventricular pacing was prioritized.

Statistical analysis was performed using SPSS version 29.0 (SPSS Inc., Chicago, IL, USA). Continuous variables were expressed as mean values with their associated standard deviations and were compared using a Student’s *t*-test. Categorical variables were analyzed using a Fisher’s exact test. Statistical significance was set at a *p*-value less than 0.05.

## 3. Results

### 3.1. Baseline Characteristics

A total of 124 patients underwent permanent pacemaker (PPM) implantation using the LBBP technique with a mean follow-up of 19.7 ± 13.3 months. In total, 90 patients were implanted with LLLs and 34 with SDLs. There was no significant difference in the baseline characteristics between the LLL and SDL groups. The average age of the patients was 70.1 ± 13.8 years old; 58.1% were male and the pre-implant LVEF was 55.6 ± 12.6%. Indications for pacing included sinus node dysfunction (40.3%), high-grade AVB (54.8%), and CRT purposes in patients with heart failure. The baseline QRS morphology of patients was normal in 54.1%; right bundle branch block (RBBB) 12.3%; RBBB with fascicular block 10.6%; LBBB 12.3%; intraventricular conduction delay (IVCD) 4.9%; alternating BBB < 1%; paced morphology from temporary pacing wire 4.9%. The average baseline QRS width was 116.3 ± 27.8 ms. These characteristics are summarized in Table 1.

### 3.2. Procedure and Success Rates

Similar rates of successful LBBP were achieved with both the LLLs and SDLs, with 82% meeting the LBB capture criteria and <1% achieving LVSP. There was no significant difference between the left ventricular activation time (LVAT) achieved in the SDLs and LLLs (Table 2). As expected, there was a significantly shorter measured LVAT when LBBAP was achieved versus deep septal pacing (69.2 ± 10.6 ms vs. 78.8 ± 12.3; *p* = 0.023) (Table 3). The group with the shortest LVAT achieved was the true LBBP group (68.6 ± 10.8 ms) (Table 4).

There was no significant difference in the total time taken between LLLs and SDLs: 97.8 ± 31.7 min vs. 109.2 ± 35.6 min. However, the fluoroscopy time was significantly longer in SDLs vs. LLLs (26.2 ± 17.7 min vs. 17.5 ± 13.0 min, respectively, *p* = 0.026). The final achieved paced QRS durations were similar in both groups with an average of 113.4 ± 8.3 ms. Interestingly, the on-table R wave sensing was significantly higher in LLLs versus SDLs (11.3 ± 6.2 mV vs. 9.0 ± 3.6 mV, *p* = 0.020). The recorded on-table impedance was also significantly higher in the LLLs compared to the SDLs (722.9 ± 290.0 ohms vs. 586.2 ± 185.4 ohms, *p* = 0.003) (Table 2).

On the first day post-implant, the sensed R wave in the LLL group was observed to be higher than in the SDLs (16.2 ± 6.8 mV vs. 10.8 ± 5.2 mV, *p* < 0.001). This persisted throughout the follow-up. A similar observation was noted in the impedance of LLLs vs. SDLs, with a higher impedance (625.8 ± 91.6 ohms vs. 562.8 ± 81.4 ohms, respectively, *p* < 0.001) 1 day post-implant up to the latest follow-up (527.0 ± 73.1 ohms vs. 459.6 ± 89.0 ohms, *p* < 0.001) (Table 5). Numerically, there was no significant difference in the LBBP thresholds between the groups. Interestingly, although there was an expected impedance drop for both groups, the drop in impedance for the SDLs was less than the LLLs, and this was consistent even at the latest device interrogation (Table 6). Numerically, there was also a larger increase in the R wave sensing value in the LLLs compared to the SDLs on the day post-implant, though this was not observed at subsequent follow-ups. Six patients in the LLL group required the use of a Medtronic Extended Hook Sheath using the “mother-and-child” technique in order to penetrate the septum successfully.

### 3.3. Complications

A total of eight device-related complications occurred with no significant differences between the LLLs and SDLs. There was one pocket hematoma; one right atrial lead fracture in the LLL group; one lead perforation on day 6 requiring a new LBBP lead; and two tamponades and two device infections. One patient in the SDL group had microdislodgement post-slitting with loss of the RSR’ pattern on pacing, and the decision was made to reposition the lead on the table. Ten patients demised, but all were unrelated to the device implant. There was no significant difference in the rates of heart failure hospitalization between the groups (Table 7). One patient from the SDL group with advanced ischemic cardiomyopathy failed an attempted LBB-optimized CRT, and only deep septal pacing was achieved. He continued to have recurrent heart failure admissions and required a left ventricular assist device to be implanted shortly after.

## 4. Discussion

Whilst HBP was an attractive option, it was imperfect. The His bundle is a small area embedded in fibrous tissue. This causes issues with sensing, high capture thresholds requiring lead revisions, and earlier generator changes [7,8,9,10]. These patients often required the implant of a right ventricular (RV) lead as a “backup” in case the HBP failed. Whilst HBP is the most physiological form of pacing, the success rate of the LBBP implant is higher [11], and the paced QRS duration is still significantly narrower than conventional RV pacing [8,9]. LBBP overcomes the limitations of HBP as it targets the larger left bundle branch area, and a site distal to the conduction block can be selected. With the lead screwed deep into the septum, a larger R wave amplitude and lower capture thresholds are achieved [10,11], giving patients excellent pacing parameters and improved battery longevity. Hence, physicians have felt more inclined to consider LBBP over HBP since the advent of this technique.

An EHRA physician survey conducted in 2023 showed that most implanters used the Medtronic 3830 SelectSecure LLL during LBBP implantation [12]. This is likely because Huang et al. introduced the idea of LBBP in 2017 using this lead [3]. Likewise, our physicians were more inclined to choose LLLs as they were the first system described in the literature and the lead of choice internationally. At the initial adoption period, no dedicated fixed sheath was available for SDLs yet, and we had to employ a self-modified technique with the Agilis Hispro [13] to expand our options for different types of patients.

It is important to note that the structure and design of SDLs and LLLs are significantly different, and this may affect their behavior within the interventricular septum (IVS). SDLs are known to be stiffer, with a larger lead body diameter (5.6 to 6 Fr), and with the stylet inserted, each turn provides a more robust torque transfer compared to LLLs [6]. SDLs also have the benefit of providing the continuous monitoring of ECG-paced morphology during lead deployment into the IVS [6,14]. However, they have a retractable helix which may result in helix retraction during deployment, a higher risk of lead damage, and acute on-table lead microdislodgement [14,15,16,17,18,19]. LLLs lack an inner lumen and have a thinner lead body of 4.1 Fr with a fixed helix. This provides stability and avoids the issue of helix retraction and reduces the risk of microdislodgement [6,15,16,17,18,19].

Like described in the literature, we had a high rate of successful LBBP implants with both SDLs and LLLs, with comparable procedure times and complications rates. There was no difference in the LVAT achieved either. The pacing impedance in SDLs was also observed to be significantly lower, like that described in the literature [14,15,16,17]. One likely reason is that SDLs have a larger diameter, and hence a lower pacing impedance is expected; pacing impedance is inversely related to the contact area and is also dependent on the length of the conductor [17]. This also explains why we continued to observe a difference in lead impedance between SDLs and LLLs on the latest device check. Although we did see a statistically significant higher on-table threshold in the SDLs, numerically they were not very different (0.9 ± 0.3 vs. 0.8 ± 0.3 mV at 0.4/0.5 ms, *p* = 0.01). The key difference was the significantly longer fluoroscopy time in the SDL vs. LLL group (26.2 ± 17.7 min vs. 17.5 ± 13.0 min, *p* = 0.026). This was because the Abbott Agilis HisPro Catheter often required attempts at reshaping to provide an extended reach and septal curve to direct the lead to an ideal position in the septum [19]. On top of this, helix retraction was also an issue, and this required further fluoroscopic guidance during the procedure.

**Figure 1:**
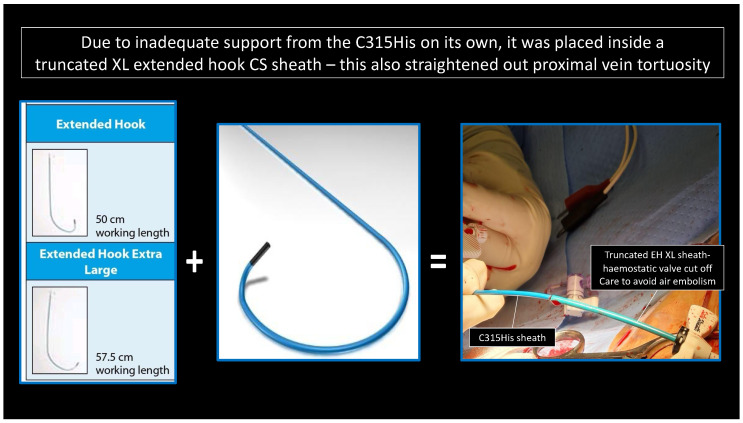
“Mother-and-child” technique: C315His and Extended Hook CS sheath-in-sheath technique.

**Figure 2:**
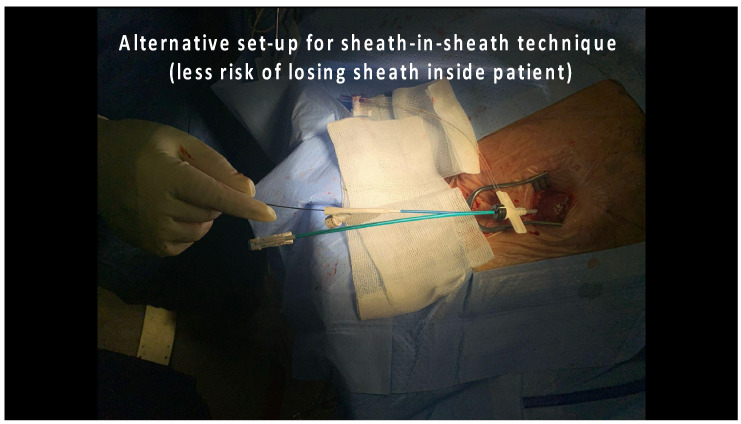
“Mother-and-child” technique: alternative modification of the Extended Hook CS sheath.

**Figure 3:**
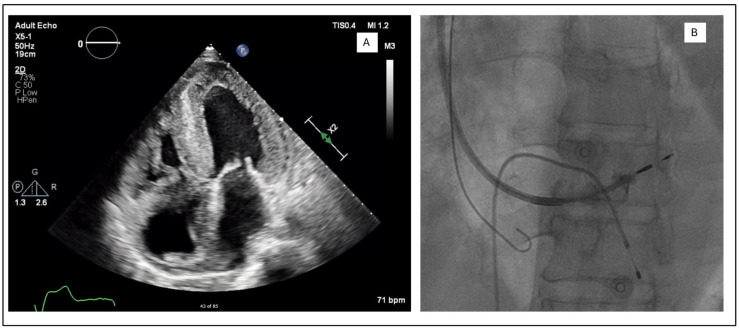
(**A**) Apical four chamber view showing biventricular wall thickening and biatrial dilatation. (**B**) Use of sheath-in-sheath technique with an extended hook to provide additional support during the deployment of the Medtronic SelectSecure 3830 lead. Venogram shows that the ring is deeply embedded in the septum.

In the LLLs, the thinner lead body and absence of a stylet, together with a less aggressive C315His sheath, made penetration in some cases harder. In this study, 6 out of 124 patients required the additional use of a self-modified Medtronic Extended Hook Sheath, which is typically used in coronary sinus cannulation (Figure 1 and Figure 2). This technique utilizes a “mother-and-child” and sheath-in-sheath technique to provide better stability, hence allowing torque to be transmitted to the lead and facilitate challenging septal penetrations. One of the patients was a gentleman who had transthyretin (ATTR) cardiac amyloidosis and developed high-grade AVB. After multiple failed attempts at penetration of the significantly thickened IVS, the above technique was utilized and the lead was successfully advanced, achieving LBBP capture (Figure 3). Perhaps if an SDL was selected for this case, we would have avoided this, and procedure time would have been reduced. Careful pre-implant planning is crucial in increasing the success of implants, and for similar patients, an SDL would have an edge over an LLL.

### Study Limitations

This was a prospective, non-randomized trial with only two tertiary centers involved. As LLLs have a first-mover advantage, the majority of physicians were more inclined to choose LLLs over SDLs, hence leading to a bias toward the implantation of LLLs. Not all the other pacing parameters like QRS transition to LVSP or selective LBBP at the threshold test were documented; hence, they could not be compared between the groups. Not all patients had ECGs performed at every follow-up visit, thus the persistence of the left bundle area pacing could not be studied. Furthermore, not all patients had an echocardiography performed post-implant. As such, long-term issues like pacing-induced cardiomyopathy and tricuspid regurgitation could not be studied.

## 5. Conclusions

LBBP is set to continue to ride the wave of popularity as it provides physiological pacing with the benefit of excellent pacing parameters. SDLs and LLLs are structurally different but share similar success and risk profiles. However, we have yet to see the long-term performance of these leads. No lead is perfect, but with careful patient selection, we can perhaps select the most suitable lead for the patient.

## Figures and Tables

**Table 1:** Baseline characteristics.

Variables	Total (*n* = 124)	LLL (*n* = 90)	SDL (*n* = 34)	*p*-Value
Age, y	70.1 ± 13.8	69.1 ± 14.3	72.9 ± 12.0	0.140
Male sex (%)	72 (58.1%)	55 (61.1%)	17 (50.0%)	0.180
IHD (%)	40 (32.3%)	28 (31.1%)	12 (35.3%)	0.405
CMP (%)	22 (17.7%)	15 (16.7%)	7 (20.6%)	0.393
ESRF (%)	5 (4.0%)	3 (3.3%)	2 (5.9%)	0.419
HTN (%)	91(73.4%)	66 (73.3%)	25 (73.5%)	0.587
HLD (%)	90 (72.6%)	63 (70.0%)	27 (79.4%)	0.207
DM (%)	52 (41.9%)	34 (37.8%)	18 (52.9%)	0.093
PAD (%)	3 (2.4%)	1 (1.1%)	2 (5.9%)	0.182
Stroke (%)	10 (8.1%)	6 (6.7%)	4 (11.8%)	0.277
PCI (%)	18 (14.5%)	11 (12.2%)	7 (20.6%)	0.184
CABG (%)	12 (9.7%)	9 (10.0%)	3 (8.8%)	0.573
VHD (%)	32 (25.8%)	23 (25.6%)	9 (26.5%)	0.543
IE (%)	3 (2.4%)	1 (1.1%)	2 (5.9%)	0.182
AF (%)	43 (34.7%)	30 (33.3%)	13 (38.2%)	0.379
Antithrombotic (%)	75 (60.5%)	56 (62.2%)	19 (55.9%)	0.329
SAPT	26	21	5	
DAPT	6	4	2	
DOAC	35	27	8	
VKA	8	4	4	
Pre-implant LVEF, %	55.6 ± 12.6	56.3 ± 11.4	53.6 ± 15.5	0.347
PPM indication (%)				
SND	50 (40.3%)	35 (38.9%)	15 (44.1%)	
AVB	68 (54.8%)	51 (56.7%)	17 (50.0%)	0.321
HF	6 (4.8%)	4 (4.4%)	2 (5.9%)	
Baseline QRS morphology (%)				0.164
Normal	66 (54.1%)	48 (53.9%)	18 (54.5%)	
RBBB	15 (12.3%)	10 (11.2%)	5 (15.2%)	
RBBB + LAFB/LPFB	13 (10.6%)	10 (11.2%)	3 (9.1%)	
LBBB	15 (12.3%)	11 (12.4%)	4 (12.1%)	
IVCD	6 (4.9%)	5 (5.6%)	1 (3.0%)	
Alternating BBB	1 (0.8%)	0	1 (3.0%)	
Dependent on TPW	6 (4.9%)	5 (4.1%)	1 (0.8%)	
Baseline QRS width, ms	116.3 ± 27.8	117.2 ± 27.6	114.1 ± 28.4	0.594

**Table 2:** Procedural details between LLLs and SDLs.

Variables	Total (*n* = 124)	LL (*n* = 90)	SDL (*n* = 34)	*p*-Value
Procedure time, min	100.8 ± 33.0	97.8 ± 31.7	109.2 ± 35.6	0.148
Fluoroscopy time	19.9 ± 14.8	17.5 ± 13.0	26.2 ± 17.7	0.026
LVAT (*n* = 123) *	70.2 ± 11.1	69.7 ± 10.7	71.2 ± 12.3	0.535
V6V1 interpeak (*n* = 37)	35.7 ± 9.0	36.1 ± 8.0	35.1 ± 10.7	0.762
On-table R wave sensing, mV	10.7 ± 5.7	11.3 ± 6.2	9.0 ± 3.6	0.020
On-table impedance, ohms	684.5 ± 271.1	722.9 ± 290.0	586.2 ± 185.4	0.003
On-table threshold, V@0.4/0.5 ms	0.8 ± 0.3	0.8 ± 0.3	0.9 ± 0.3	0.010
Paced QRSd, ms	113.4 ± 8.3	113.5 ± 8.4	113.1 ± 8.1	0.852
Type of capture				
LBBP	102	74	28	0.607
LVSP	10	8	2	
DSP	12	8	4	

* One data point missing in the measured LVAT due to corrupted data in the computer system.

**Table 3:** Difference in LVAT between LBBAP and DSP.

Variables	Total (*n* = 124)	LBBAP * (*n* = 111)	DSP (*n* = 12)	*p*-Value
LVAT (*n* = 123)	70.2 ± 11.1	69.2 ± 10.6	78.8 ± 12.3	0.023

* LBBAP denotes both LBBP and LVSP.

**Table 4:** Difference in LVAT between LBBP and LVSP.

Variables	Total (*n* = 111)	LBBP (*n* = 101)	LVSP (*n* = 10)	*p*-Value
LVAT (*n* = 111)	69.2 ± 10.6	68.6 ± 10.8	75.2 ± 10.4	0.012

**Table 5:** LBB lead parameters between LLLs and SDLs.

Variables	Missing	Total (*n* = 124)	LLL (*n* = 90)	SDL (*n* = 34)	*p*-Value
POD1 R wave sensing, mV	20	14.6 ± 6.8	16.2 ± 6.8	10.8 ± 5.2	<0.001
POD1 impedance, ohms	1	608.9 ± 93.0	625.8 ± 91.6	562.8 ± 81.4	<0.001
POD1 threshold, V@0.4 ms	0	0.5 ± 0.2	0.5 ± 0.2	0.6 ± 0.2	0.024
POD1 Paced QRSd, ms	28	117.2 ± 16.6	119.4 ± 10.1	111.5 ± 26.7	0.155
3 m R wave sensing	18	15.6 ± 7.0	17.0 ± 6.9	11.8 ± 5.8	<0.001
3 m impedance, ohms	5	557.5 ± 112.3	549.6 ± 99.0	470.1 ± 124.4	0.002
3 m threshold, V@0.4 ms	7	0.8 ± 0.2	0.8 ± 0.2	0.9 ± 0.3	0.012
3 m PacedQRSd, ms	72	117.1 ± 15.6	119.2 ± 16.0	113.9 ± 14.8	0.220
Latest R wave sensing	50	13.1 ± 7.1	14.3 ± 7.2	9.6 ± 5.4	0.005
Latest impedance, ohms	5	508.5 ± 83.1	527.0 ± 73.1	459.6 ± 89.0	<0.001
Latest threshold, V@0.4 ms	11	0.9 ± 0.2	0.9 ± 0.2	0.9 ± 0.2	0.188
Latest PacedQRSd, ms	67	118.9 ± 19.2	120.6 ± 18.1	113.9 ± 21.6	0.299

**Table 6:** LLLs vs. SDLs—Comparing change in impedance and sensing during follow-up.

Independent Samples Test (Equal Variances Assumed)							
LLL vs. SDL	*t*-Test for Equality of Means						
	t	df	Sig. (2-Tailed)	Mean Difference	Std. Error Difference	95% Confidence Interval of the Difference	
						Lower	Upper
POD1 Impedance	−3.095	108	0.003	−108.82523	35.16569	−178.52972	−39.12074
3-months Impedance	−3.296	105	0.001	−118.18718	35.85860	−189.28817	−47.08620
Latest Impedance	−3.353	104	0.001	−117.60258	35.07275	−187.15316	−48.05200
POD 1 Sensing	1.985	88	0.050	2.69817	1.35905	−0.00265	5.39900
3-months Sensing	1.657	90	0.101	2.56948	1.55055	−0.51096	5.64992
Latest sensing	1.506	61	0.137	3.00787	1.99675	−0.98489	7.00063

**Table 7:** Complications and incidence of heart failure hospitalizations.

Variables	Total (*n* = 124)	LLL (*n* = 90)	SDL (*n* = 24)	*p*-Value
HF hospitalisation	13 (10.5%)	9 (10.0%)	4 (11.8%)	0.501
Pocket hematoma	1 (0.8%)	0 (0.0%)	1 (2.9%)	0.276
Lead dislodgement	0 (0.0%)	0 (0.0%)	0 (0.0%)	NA
Lead fracture	1 (0.8%)	1 (1.1%)	0 (0.0%)	0.724
Lead extraction	2 (1.6%)	1 (1.1%)	1 (2.9%)	0.478
Tamponade	2 (1.6%)	1 (1.1%)	1 (2.9%)	0.478
CIED infection	2 (1.6%)	0 (0.0%)	2 (5.9%)	0.075
Pneumothorax	0 (0.0%)	0 (0.0%)	0 (0.0%)	NA
All-cause mortality	10 (8.1%)	7 (7.8%)	3 (8.8%)	0.551

## Data Availability

The original contributions presented in this study are included in the article.
